# Krüppel-Like Factor 8 (KLF8) Is Expressed in Gliomas of Different WHO Grades and Is Essential for Tumor Cell Proliferation

**DOI:** 10.1371/journal.pone.0030429

**Published:** 2012-01-19

**Authors:** Oliver Schnell, Alexander Romagna, Irene Jaehnert, Valerie Albrecht, Sabina Eigenbrod, Kathrin Juerchott, Hans Kretzschmar, Jörg-Christian Tonn, Christian Schichor

**Affiliations:** 1 Department of Neurosurgery, Klinikum der Ludwig-Maximilians-Universität München, Campus Grosshadern, Munich, Germany; 2 Center for Neuropathology and Prion Research, Ludwig Maximilians-Universität München, Munich, Germany; 3 Max Planck Institute of Molecular Plant Physiology, Wissenschaftspark Golm, Potsdam, Germany; Virginia Commonwealth University, United States of America

## Abstract

Krüppel-like factor 8 (KLF8) has only recently been identified to be involved in tumor cell proliferation and invasion of several different tumor entities like renal cell carcinoma, hepatocellular carcinoma and breast cancer. In the present study, we show for the first time the expression of KLF8 in gliomas of different WHO grades and its functional impact on glioma cell proliferation. In order to get information about KLF8-mRNA regulation qPCR was performed and did not reveal any significant difference in samples (n = 10 each) of non-neoplastic brain (NNB), low-grade gliomas (LGG, WHO°II) and glioblastomas (GBM, WHO°IV). Immunohistochemistry of tissue samples (n = 7 LGG, 11 AA and 12 GBM) did not show any significant difference in the fraction of KLF8-immunopositive cells of all analyzed cells in LGG (87%), AA (80%) or GBM (89%). Tissue samples from cerebral breast cancer metastasis, meningiomas but also non-neoplastic brain demonstrated comparable relative cell counts as well. Moreover, there was no correlation between KLF8 expression and the expression pattern of the assumed proliferation marker Ki67, which showed high variability between different tumor grade (9% (LGG), 6% (AA) and 15% (GBM) of Ki67-immunopositive cells). Densitometric analysis of Western blotting revealed that the relative amount of KLF8-protein did also not differ between the highly aggressive and proliferative GBM (1.05) compared to LGG (0.93; p<0.05, studens t-test). As demonstrated for some other non-glial cancer entities, KLF8-knockdown by shRNA in U87-MG cells confirmed its functional relevance, leading to an almost complete loss of tumor cell proliferation. Selective blocking of KLF8 might represent a novel anti-proliferative treatment strategy for malignant gliomas. Yet, its simultaneous expression in non-proliferating tissues could hamper this approach.

## Introduction

The zinc finger-containing transcription factor Krüppel-like factor 8 (KLF8) [1] has first been accounted to act as a downstream molecule for integrin-dependent FAK-signaling in mouse fibroblast NIH3T3 cells [2]. Zhao and colleagues reported that stimulation of the focal adhesion kinase (FAK) led to a FAK/Src/PI-3K triggered KLF8 binding to cyclin D1 and consecutive entrance into the replication phase of the cell cycle in these cells. In different solid cancers outside the central nervous system (CNS), FAK has been implicated as a leading signal transduction molecule for crucial integrin-dependent steps of adhesion and proliferation as well as invasion and migration of tumor cells [3], [4].

These attributes would render KLF8 also a very interesting target for novel treatment approaches in GBM. Unlike the inhibitory effect achieved by blocking of upstream molecules as for example integrin α_v_β_3_, a more downstream interference might be less susceptible against compensatory “back-up” mechanisms of malignant tumor cells. Moreover, recent findings support the importance of KLF8 in malignant cell transformation and tumor growth of several different non-CNS tumors [5], [6], [7], [8]. KLF8 protein and mRNA expression was not only significantly higher in renal cell carcinoma (RCC) than in non-neoplastic renal tissue, inhibition of KLF8 via siRNA could also induce cell apoptosis in vitro and reduced tumor growth in vivo [7]. Furthermore, KLF8 overexpression was demonstrated in highly metastatic or recurrent hepatocellular carcinomas (HCC). In this study, KLF8 could even be linked with clinical data and revealed to be a significant predictor for overall and progression-free survival [8]. Only recently additional data was presented, which identified KLF8 as an activator for matrix metalloproteinase 9 (MMP9), leading to enhanced tumor invasion of human breast cancer [9].

This study was performed in order to assess for the first time expression and functional impact of KLF8, a member of the Krüppel-like transcription factor family, in primary brain tumors. Extended knowledge of proliferation related transcription factors like KLF8 might help to identify target structures for the development of new therapeutic drugs in the complex and demanding treatment strategies of glial brain tumors.

## Methods

### Patients and tissue samples

Tissue samples were obtained from patients with newly diagnosed gliomas. Clinical data of these patients are summarized in supplementary Table S1. Histological diagnosis was performed on paraffin embedded sections according to the World Health Organization (WHO) criteria [10]. Histologically confirmed low-grade astrocytomas (LGG; WHO°II, n = 10), anaplastic astrocytomas (AA, WHO°III, n = 11) and glioblastomas (GBM; WHO°IV, n = 12), as well as non-neoplastic brain tissue (NNB, n = 10) obtained from patients with epileptic focus resection were included in this study. Additionally, meningioma, cerebral breast cancer metastasis and renal cell carcinoma (n = 3 each) were analyzed. Written informed consent was obtained from all patients according to the study approval of the ethics committee of the Ludwig-Maximilians-Universität München. Tumor tissue samples were either paraffin embedded for immunohistochemistry or snap frozen and homogenized for PCR and Western blot analysis.

### PCR

RNA from snap frozen tumor samples (n = 10 NNB, LGG and GBM) or U87-MG cells (5×10^5^) was isolated with the RNeasy® Mini-Kit (QIAGEN GmbH) according to manufacturers̀ instructions. The RNA (a total of 0.2 µg for each sample) was then reverse transcribed using the QuantiTect® Reverse Transcription Kit (QIAGEN GmbH). The semi-quantitative PCR was performed using the LightCycler system (Roche Diagnostics GmbH) according to manufacturerś instructions by using QuantiFast SYBR® Green PCR Kit (QIAGEN GmbH). In brief, 2 µl cDNA was used for amplification in a total volume of 20 µl per capillary. The following primer pair was used: KLF8 fwd: 5′TGG AAC CAG TGG ACC TCT CC3′; KLF8 rev: 5′-ATC TGC TGG CTA CCA GTG CT-3′. The fluorescent signals obtained were used to calculate the relative amount of template DNA. Signals for KLF8 were normalized against the TBP housekeeping gene (for U87-MG) and HPRT1 and RRN18S housekeeping genes (for tissue) (QuantiTect® Primer Assays, QIAGEN).

### Histology and Immunohistochemistry

The histological diagnosis was made by the members of the local histopathological conference according to the 2007 WHO classification of tumors of the central nervous system. For histopathological examination, samples were fixed with 4% buffered formalin (Fisher Scientific GmbH) and embedded in paraffin. Tumor morphology in 2 µm paraffin sections was visualized using hematoxylin and eosin staining. For immunohistochemistry, sections were subjected to standardized staining on a benchmark staining machine with a 3,3′-diaminobenzidine (DAB) detection system according to the manufacturerś instructions (Ventana Medical Systems). Primary antibodies against the transcription molecule KLF8 (1∶100, AV31533, Sigma) were used for detection of human KLF8. Proliferation activity in the respective areas was determined using anti-human Ki67 antibody (mouse monoclonal, clone MIB-1, Dako). For quantification of KLF8 and Ki67, first a representative area was selected in the KLF8 stained section and the corresponding area was identified in consecutive Ki67-stained section. Then, for all cases representative pictures (400× magnification, area: 35525 µm^2^ for each area) of the selected regions in the KLF8 and Ki67 sections were taken with a BX50 Olympus microscope. Cell count was performed using Cell D Software according to the manufacturerś instructions. All immunohistochemical staining procedures and antibody concentrations were adjusted to avoid overstaining (saturation), understaining (beyond detection limit) or non-specific background artifacts as described elsewhere [11]. Negative controls were performed by omitting the primary antibody, tissue samples from a renal parenchyma served as a positive control (Figure S1 A).

For immunocytochemistry, malignant glioma cell line U87-MG was cultured in vitronectin-coated (2%) CultureSlides (BD Falcon GmbH) and fixed with 4% paraformaldehyde (20°C, 15 min). For detection of nuclear proteins, permeabilization with methanol (−20°C, 20 sec) was performed. After incubation with primary antibody against KLF8 (1∶100, H00011279-B01, Abnova GmbH) diluted in antibody diluent, slides were treated with biotinylated secondary antibody (1∶750, BA-2000, Vector Laboratories, Inc.) for 15 min followed by alkaline phosphatase conjugated streptavidin (1∶1000, SA-5100, Vector Laboratories, Inc.) for 30 min. Visualization was performed using 5-bromo-4-chloro-3-indoyl phosphate p-toluidine salt and p-nitro blue tetrazolium chloride solution (BCIP/NBT, Vector Laboratories, Inc.), followed by counterstaining with eosine (Sigma-Aldrich Chemie GmbH). Slides were dehydrated by an ascending ethanol sequence (70%, 96%, 100%) and histol (Carl Roth GmbH und Co. KG) and finally covered (Figure S1 B). Negative controls were performed by omitting the primary antibody.

### Western Blot

Western blotting was performed from tissue samples of NNB, LGG and GBM (n = 10 each) as described previously with only slight modifications [11]. Snap frozen tissue sections were minced and homogenized on ice under addition of NP-40 lysis buffer (10 mM Tris/HCl, 150 mM NaCl, 10% Glycerol, 0.5% Nonidet P-40 (Roche Diagnostics GmbH), 2 mM EDTA pH 8.0 (Sigma-Aldrich Chemie GmbH) containing 1 mmol/l DTT, 0,5 mmol/l PMSF (Sigma-Aldrich Chemie GmbH), PIC (Sigma-Aldrich Chemie GmbH), Phosphatase Inhibitor Cocktail 1 and 2 (Sigma-Aldrich Chemie GmbH)). After incubation in lysis buffer for 15 min on ice, lysates were centrifuged at 14000 x g, 4°C for 10 min. The quantification of protein in the supernatant was performed via Bradford protein assay (BioRad Laboratories GmbH). Briefly, equal amounts of protein (30 µg) were separated by SDS-Page (10%) followed by transfer to PVDF membrane (BioRad Laboratories GmbH). After Blocking with 1 x Roti-Block (Carl Roth GmbH & Co. KG) for 2 hrs, the membrane was incubated with primary antibody (1∶1000, SAB2101277, Sigma-Aldrich Chemie GmbH) diluted in 1 x Roti-Block at 4°C over night. The membrane was washed in TBS-Tween 20 (Sigma-Aldrich Chemie GmbH) for three times, incubated with secondary antibody (1∶10000, 170-5046, BioRad Laboratories GmbH) and again washed for three times. Proteins were visualized by enhanced chemiluminiscence detection system (Pierce ECL Western Blotting Substrate, Thermo Fisher Scientific Germany Ltd. & Co. KG). Equal loading was confirmed by use of a mouse anti-β-actin antibody (1∶1000, sc-47778, Santa Cruz Biotechnology, Inc.).

Semiquantitative analysis of Western blot results was performed by densitometric measurements with the software ImageJ (Image Processing and Analysis in Java) by using a protein ladder (Invitrogen GmbH) for calibration of the molecular weights. Optical Density was normalized onto the house keeping gene β-actin, equalling 1.0. To compare different membranes, equal aliquots of a protein lysate of U87-MG cells were processed on each membrane.

### Cell culture

The human glioblastoma cell line U87-MG was cultured in Dulbecco's modified Eagle medium (DMEM, Biochrom AG) containing minimum essential medium (MEM) with non-essential amino acids (NEAA, Invitrogen GmbH), fetal bovine serum (FBS, 10%, Biochrom AG) and additives (penicillin-streptomycin-glutamine, Invitrogen GmbH). Cells were cultured on vitronectin-coated (2%; Sigma-Aldrich GmbH) plastic flasks unto subconfluency for all purposes (TPP AG).

### KLF8-knockdown via shRNA transfection

Assay ready U87-MG KLF8-knockdown as well as scrambled shRNA transfected cells were purchased from Sirion Biotech, Martinsried, Germany. Therefore, U87-MG cells from our laboratory stock were transfected with adenoviral vectors by Q-tech silencing technique as described elsewhere [12]. Shortly, double stranded sh-oligonucleotide cassettes were cloned under the control of human U6-promoter into plasmid A. The U6-shDNA portion was then transferred by recombination into the backbone of a standard E1/E3 deleted, replication-deficient Ad5 vector which was contained in plasmid B. Subsequent virus rescue and production was carried out in HEK293 cells. Virus purification was performed chromatographically using commercially available adenovirus purification kits. Infectious virus titers were determined with Adeno-XTM Rapid Titer Kit (Clontech Laboratories, Inc.). For seeding of Q-tech cells our standard medium (c.f. cell culture) was used.

For verification of KLF8-knockdown, cells were monitored over a period of 72 hrs after cell seeding. Maximum level of KLF8-knockdown within the test period was found to be >90% 72 hrs after seeding as quantified by qPCR. Remaining protein expression after successful knockdown of KLF8-mRNA was analyzed by Western Blot of the nuclear protein fraction in all transfected cells. Therefore, cells were washed twice with 1 x PBS and first lysed to remove cytoplasma proteins with buffer A [10 mM HEPES pH 7.9; 10 mM KCl; 1.5 mM MgCl_2_ x 6 H_2_O; 2 mM DTT; 10 µg/ml Aprotinin; 0.5 mM PMSF] for 10 min on ice followed by treatment with ultrasound in a Bandelin Sonorex Super RK 52 H device (HFfrequency 35 kHz, HFpower 60/120 W) for 5 sec, short incubation on ice and again treatment with ultrasound for 3 sec. After centrifugation at 14000 x g, 4°C for 10 sec, equal amount of 60% Glycerol was added to the supernatant. For extraction of nuclear fraction, same volume of buffer B [20 mM HEPES pH 7.9; 25% Glycerol; 0.42 M NaCl; 1.5 mM MgCl_2_ x 6 H_2_O; 0.2 mM EDTH pH 8,0; 2 mM DTT; 10 µg/ml Aprotinin; 0,5 mM PMSF] was added to the pellet followed by incubation on ice for 45 min. After centrifugation for 10 min at 8000 x g, 4°C the same volume of buffer C [20 mM HEPES pH 7,9; 20% Glycerol; 0,1 mM KCl; 0,05 mM EDTH pH 8,0; 2 µl/ml Igepal] was added.

### Proliferation Assay

To confirm functional relevance of KLF8-knockdown in the transfected U87-MG cells, a proliferation assay was performed. Scrambled shRNA-transfected U87-MG cells as well as KLF8-knockdown U87-MG cells were seeded at passage 1 after transfection in equal amounts (each 1.0×10^4^ cells/cm^2^) in vitronectin coated 24-well plates and cultured in DMEM medium for as long as 4 days after seeding. Each day, cell counts were measured by direct cell counting of vital cells via trypan blue staining (Sigma-Aldrich Chemie GmbH). Total cell counts were documented for each well. After four repetitions with equal experimental conditions, cell counts were compared and mean values as well as standard deviations were calculated.

### Statistical Analysis

Further statistical analysis of the data was performed with R (www.r-project.org). Since the experimental design included repeated measurements for two experimental groups (KLF8- knockdown, scarmbled shRNA-transfected) at different time points (days 1, 2, 3, 4), a two-way ANOVA (library “stats”, function “aov”) was used to investigate the impact of the two parameters (experimental group, time point) on the number of the cells simultaneously. Both parameters, experimental group and time point, as well as their interaction were identified as significant. Therefore, the 8 groups of replicate samples (2 experimental groups x 4 time points) were tested pairwise with t-Tests. “Holm” correction was applied to correct for multiple testing. (library “stats”, function “pairwise.t.test”).

## Results

### KLF8-mRNA is expressed in glioma tissue of different grades as well as non-neoplastic brain

**Figure 1:**
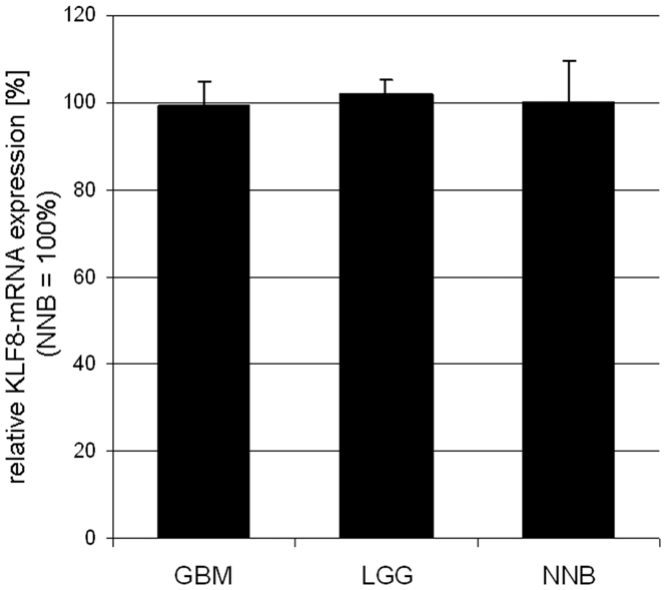
Quantitative PCR. Samples of low-grade gliomas LGG and GBM (n = 10 each) were analyzed for KLF8-mRNA regulation. Compared to non-neoplastic brain (set as 100%), qPCR did not show any significant difference in the amount of KLF8-mRNA in LGG (97.1%) and GBM (99.3%).

Compared to non-neoplastic brain, qPCR revealed no significant difference in KLF8-mRNA expression in lysates of low-grade gliomas (97.1%, relative expression) as well as glioblastomas (99.3%) (Figure 1).

### Expression of KLF8 is prominent in tumor cells of high- and low grade gliomas but does not correlate with Ki67 expression

**Figure 2:**
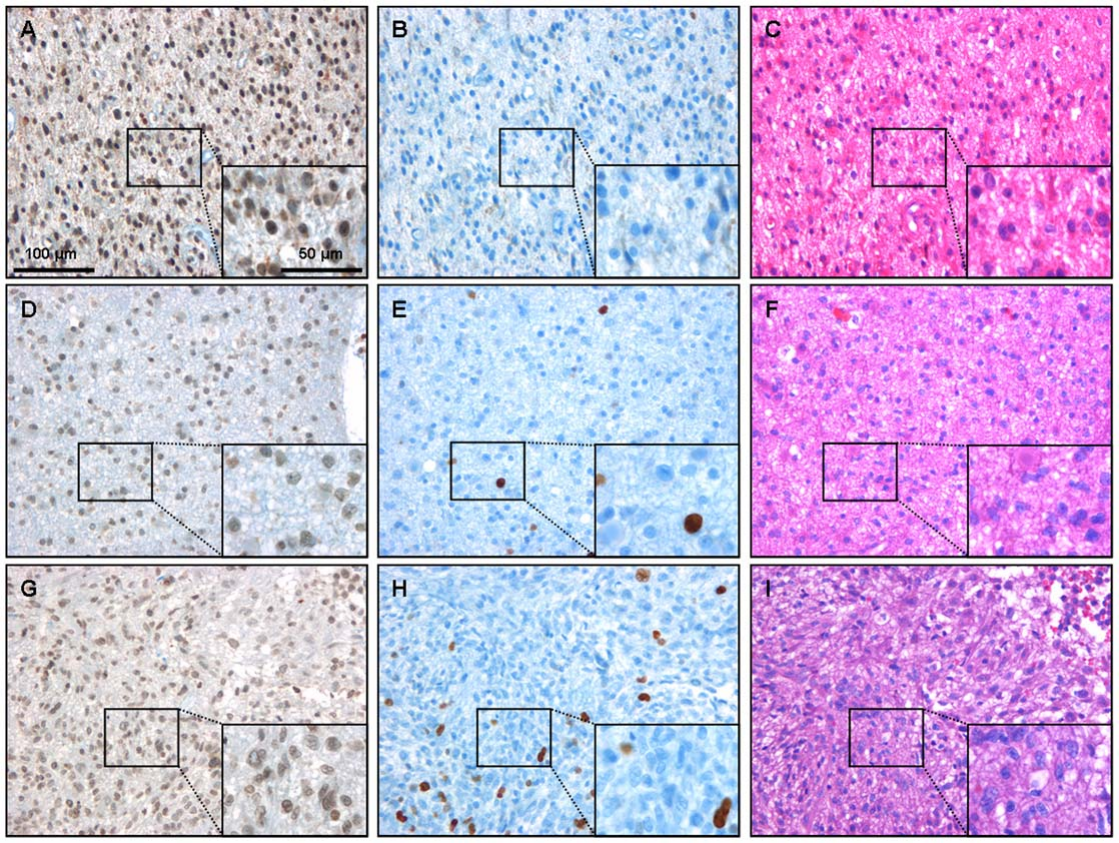
Immunohistochemistry for KLF8 and Ki67 in gliomas of different WHO-grades. Immunohistochemistry for KLF8 demonstrates that gliomas of different WHO-grades (A: °II, D: °III, G: °IV) show expression of the transcription factor. The expression is ubiquitous in the tissue and shows no grade dependency. Higher magnification demonstrates that KLF8 immunopositive staining is mainly visible in the nuclei but also present in the cytoplasm (Figure 3). Proliferation marker Ki67 is strongly expressed in nuclei of a small population of cells in the same tumors (B: °II, E: °III, H: °IV). Routine HE staining was performed on paraffin embedded sections of all tumor tissue samples (C: °II, F: °III, I: °IV). Scale bars as indicated. For cell count analysis of KLF8 and Ki67 see Table S2.

Immunohistochemical staining for KLF8 demonstrated abundant protein expression in glial tumors of different WHO grades (Figure 2). Relative cell counts of KLF8-immunopositive cells were obtained as percentage of the total number of cells within the same areas as displayed in supplementary Table S2. There was no significant difference in calculated relative cell counts of KLF8-positive cells in LGG (86%), AA (80%) and GBM (89%).

Even if KLF8 is a putative proliferation marker in other non-glial tumor entities, not all KLF8 immunopositive glioma cells did also stain positive for Ki67, which is a widely used proliferation marker in glial tumors. However, Ki67 protein expression itself only weakly correlated with tumor grading as revealed by relative cell counts of adjacent sections from the same tumor areas (Figure 2). The median Ki67 index was 9% (range: 0–39%) in the LGG, 6% (0–12%) in the AA and 16% (1–46%) in the highly proliferative GBM (Table S2).

**Figure 3:**
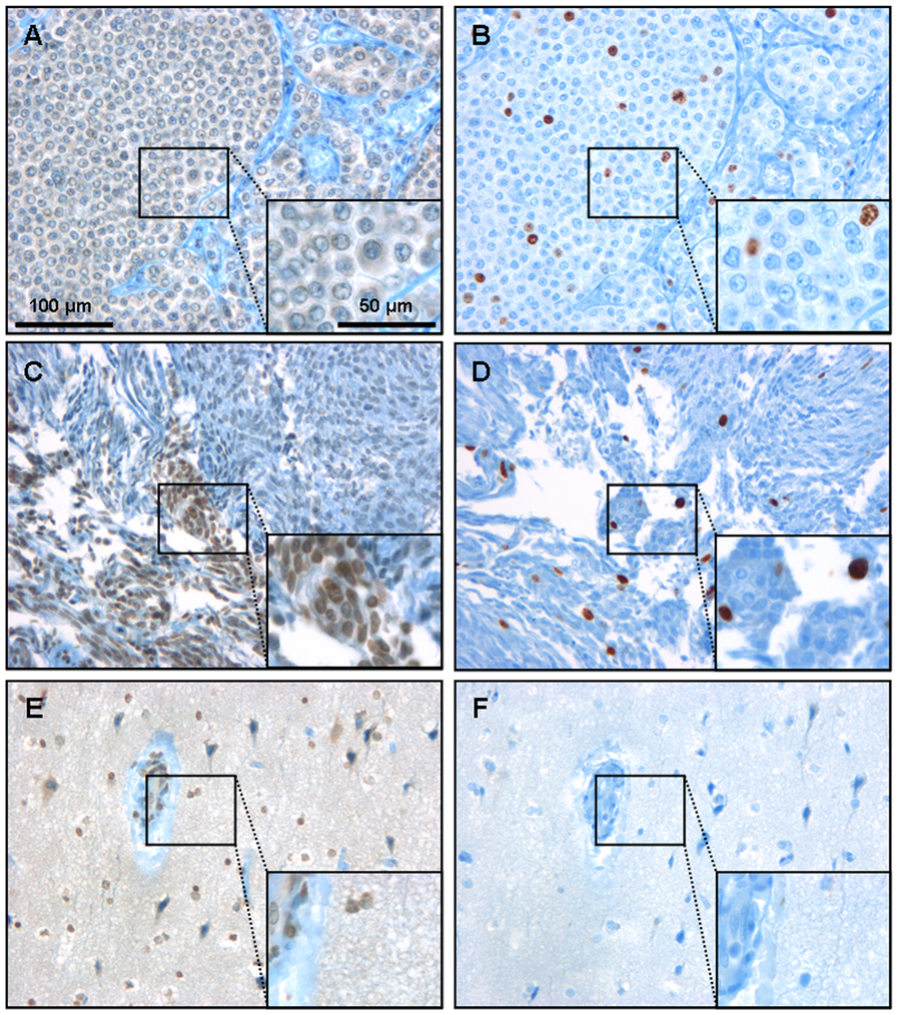
KLF8 and Ki67 expression in non-CNS tumors. KLF8 (A, C, E) and Ki67 (B, D, F) protein expression was analyzed in some additional tissue samples of breast cancer metastasis (A, B), meningioma (C, D) and non-neoplastic brain (E, F). All tissue samples analyzed confirmed ubiquitous expression of this transcription molecule, irrespective of the assumed proliferation rate.

Independent expression of Ki67 and KLF8 was also demonstrated in other tissues. Samples from breast cancer metastasis, meningioma and non-neoplastic brain showed ubiquitous expression of KLF8, irrespective of the assumed proliferation rate (Figure 3). Renal parenchyma served as a positive control for KLF8 expression (Figure S1 A).

### Quantitative analysis reveals comparable relative KLF8 protein expression in gliomas of different WHO grades

**Figure 4:**
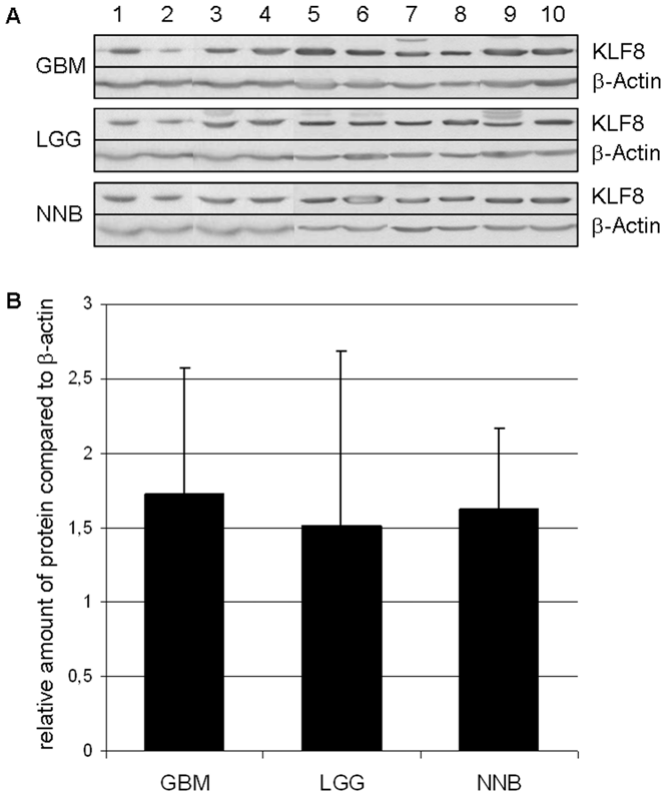
Western blot and densitometric analysis of KLF8 Western blots in gliomas. Western blot was performed for samples from gliomas of different WHO grades (GBM °IV, LGG °II) as well as non-neoplastic brain (NNB) (A). Densitometric analysis of the Western blot revealed no significant difference in expression of the transcription factor KLF8 in GBM compared to LGG and non-neoplastic brain samples. Protein expression was normalized to the house-keeping gene β-actin (set as 1.0, B).

Densitometric analysis of Western blot experiments revealed an overall density of KLF8 (36.89 kDa) of 1.72±0.85 in GBM compared to 1.52±1.16 in LGG thus indicating that KLF8 protein is expressed without significant difference (Figure 4). Hence, Western Blot quantification confirms similar relative KLF8 protein expression also in non-neoplastic brain (1.63±0.54).

### KLF8-knockdown terminates proliferation in U87-MG

**Figure 5:**
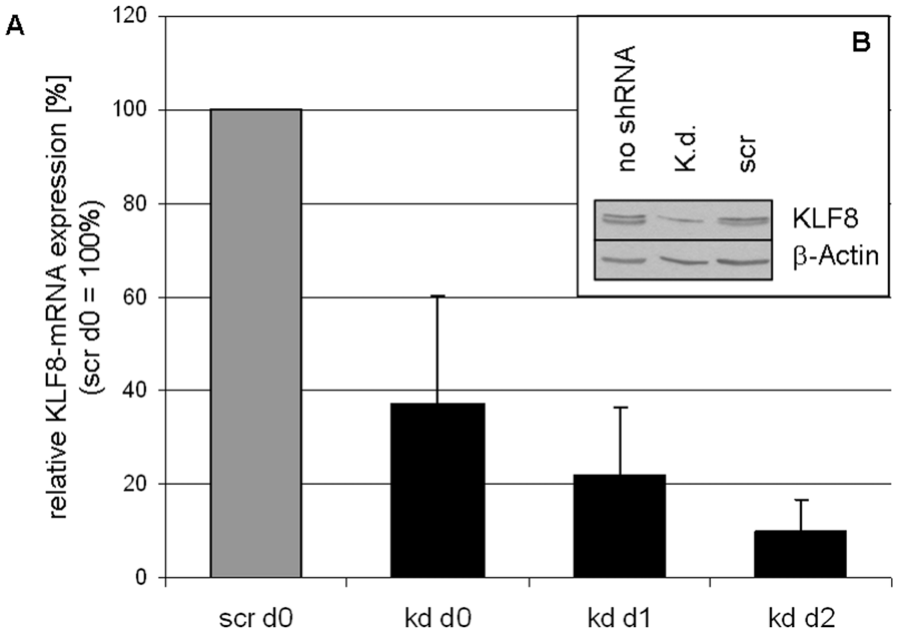
PCR and WB of KLF8 after shRNA knockdown in U87-MG. (A) U87-MG cells transfected with either scrambled (scr) or KLF8-shRNA (kd) were cultivated for up to 3 days (day 0 = day of seeding). Cells were harvested every 24 hrs and RNA was isolated, transcribed into cDNA and amplified by qPCR; data were normalized relative to levels of the house keeping gene TBP. Semi-quantitative qPCR displayed a clear knock-down in KLF8 expression already 48 hrs after transfection (day 0). Expression levels decreased to about 10% in KLF8-shRNA treated cells compared to cells treated with scrambled shRNA on day 2 after seeding. (B) Subsequent Western Blot analysis of the nuclear fraction of KLF8-kd U87-MG cells on day 4 after seeding revealed that KLF8 protein was still detectable in all transfected U87-MG cells but only to a small extent in the KLF8-knockdown cells indicating that shRNA-knockdown was successful in these transfected cells in concordance with the qPCR results (Figure 5A).

Since KLF8 has been demonstrated to be essential in proliferation of non-glial tumors, we selectively inhibited KLF8 by shRNA transfection in U87-MG glioma cell line (detected positive for KLF8, Figure S1 B). This led to an almost complete knockdown of KLF8-mRNA expression as quantified by qPCR (Figure 5 A) and reduced KLF8 protein expression as demonstrated by Western Blot analysis (Figure 5 B) in U87-MG.

**Figure 6:**
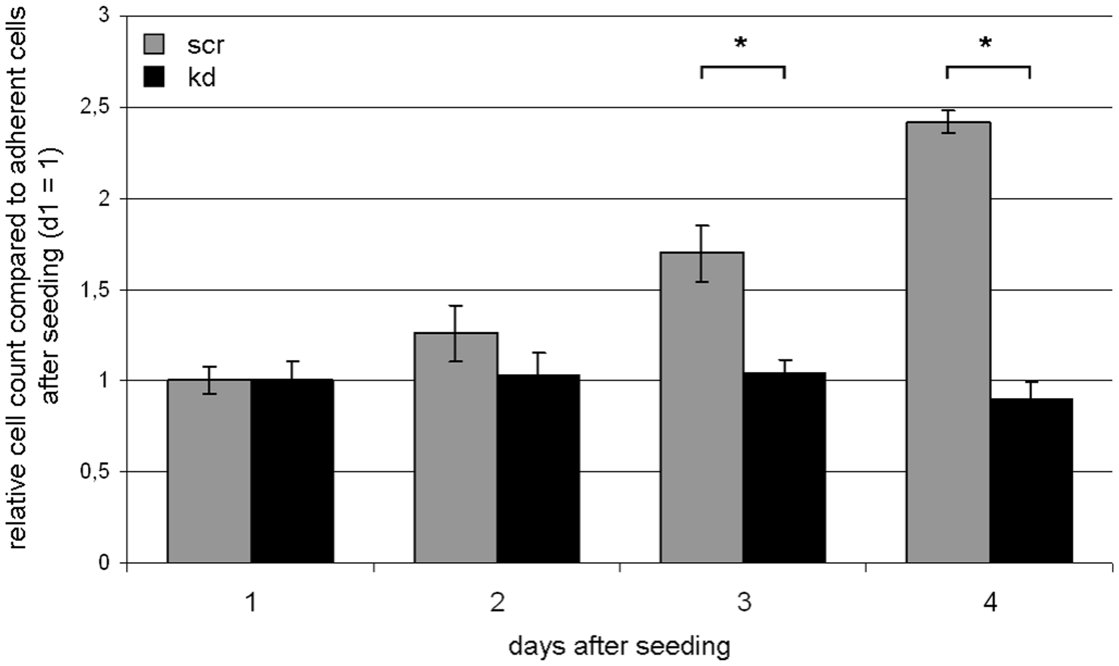
Proliferation of U87-MG in KLF8 shRNA knockdown as well as scrambled shRNA control cells. U87-MG cells were transfected with KLF8-shRNA or the scramble control, as indicated. Proliferation rate was high in the scrambled control cells during the whole time course after seeding. In contrast, KLF8-knockdown cells did show only minimal proliferation at day 2 after seeding but ceased further proliferation from day 3 on. Asterisk indicates significant difference (Mann-Whitney U test; p<0.05).

Proliferation assay of U87-MG transfected with KLF8 shRNA or scrambled shRNA revealed significant inhibition over a period of 3 days after seeding as outlined in figure 6 (Figure 6).

In order to investigate the significance of this observation, two-way ANOVA was used which analyzes the influence of the two parameters (experimental group, time point). Both parameters (experimental group, time point) as well as their interaction (p-Values: 3.2e-13, 1.0e-10, 3.5e-12) were identified as significant. Therefore, the groups were further tested pairwise with t-Tests followed by a correction for multiple testing. The scrambled shRNA-transfected cells showed proliferation with significantly increasing cell numbers from day to day (p-Values: 0.042, 1.6e-04, 5.8e-08). In contrast, there was no statistically significant difference at a p-Value ≤ 0.05 between the cell numbers for KLF8 shRNA transfected cells measured on days 1, 2, 3 and 4 (p-Values: 1, 1, 0.8) which indicates no proliferation in KLF8 shRNA-knockdown cells during the whole period analyzed.

## Discussion

Krüppel-like factor 8 (KLF8) has been demonstrated to mediate cell cycle progression downstream of focal adhesion kinase (FAK) by upregulating cyclin D1. Enhanced expression of KLF8 has been shown in several types of non-CNS tumor cells and primary tumor tissues [6] where it has been associated with malignancy. Although this might implicate similarities in glial tumors, detailed knowledge about morphological KLF8 expression in gliomas is still lacking.

Our immunohistochemical and Western blot studies demonstrate for the first time that KLF8, a pivotal transcription factor for cell cycle proliferation, is expressed in gliomas of different WHO grades (Figure 2). However, no significant difference was seen between glioma dignities both on protein level (immunohistochemistry, Western blot analysis) or mRNA level (PCR). Even if KLF8 has been depicted an ubiquitous factor [1], this is surprising since other highly proliferative non-CNS tumors such as recurrent hepatocellular carcinomas or renal cell carcinomas demonstrated significant overexpression of this signaling molecule [8]. On the other hand, these highly aggressive non-CNS tumors have mesenchymal cell origin and KLF8 has also been shown to trigger epithelial-to-mesenchymal transition [13], which might in part explain overexpression of KLF8 in these tumors.

Since KLF8 has been linked to proliferative activity of non-CNS tumors, we additionally stained consecutive sections with the widely used proliferative marker Ki67 [14]. While KLF8 was expressed in almost all glial tumors, only a limited fraction of the tumor cells were found to be immunopositive for the Ki67. Despite a tendency towards a grade dependent expression between GBM and LGG, this was not supported by the AA which had an even lower proliferative index than the LGG in our series (Table S2). Moreover, tumor-grade dependent groups were heterogeneous with regard to Ki67 expression. This inconsistent correlation with the histological malignancy has already been found by other groups, [15], [16], even if Ki67 is often used to quantify the proliferative potential of gliomas in the clinical setting [14], [17]. Thus, our results show no correlation of Ki67 with KLF8 expression and as described earlier, Ki67 should therefore be interpreted with caution in the evaluation of proliferative activity of glial tumors of all WHO grades.

Even if functional relevance of markers like Ki67 or KLF8 does not necessarily depend on protein or mRNA upregulation, deficient expression might still have an impact on cell proliferation. Hence, we subjected U87-MG cells to shRNA-knockdown of KLF8, which led to a significant time dependent impairment in their proliferation, providing first evidence for the potency of this transcription factor in human gliomas. This is in line with previous reports where inhibition of endogenous KLF8 by siRNA reduced cell cycle progression in NIH3T3 mouse fibroblasts [2]. By targeting the downstream transcription molecule KLF8, we tried to exclude compensatory pathways which might counteract treatment effects on an upstream level. Yet, there is still a multitude of KLF8-related posttranscriptional regulations as described lately in non-malignant tissues [18], [19], [20]; e.g. modulation of KLF8 transcriptional activity by co-activator proteins or by modification via acetylation [13]. It now has to be elucidated whether KLF8 regulation follows similar mechanisms in glioma models in order to identify possible new key molecules.

### Conclusion

In summary, we have identified Krüppel-like factor 8 expression in the tumor parenchyma of human gliomas of different WHO grades without quantitative correlation to tumor grade or Ki67 expression. Inhibition of this potent transcription factor led to an almost complete loss of glioma cell proliferation in vitro, but its ubiquitous expression might counteract KLF8-targeting in malignant gliomas as a future antiproliferative strategy. Nevertheless, this work significantly advances our knowledge on glioma specific KLF8 expression patterns but independent functional relevance. This study also provides information that will be useful in the future in order to study or target KLF8-activated gene-specific cellular functions in brain tumors.

## Supporting Information

Table S1 **Relevant clinical data from patients with gliomas of different WHO grades.** Histologically confirmed low-grade astrocytomas (LGG; WHO°II), anaplastic astrocytomas (AA, WHO°III) and glioblastomas (GBM; WHO°IV) from both genders (male/female) were included in our study. Tumor tissue was subjected to the different analysis as indicated: IHC  =  immunohistochemistry, WB  =  Western blot, PCR  =  polymerase chain reaction.
(TIF)

Table S2 **Cell count analysis of KLF8 and Ki67 in gliomas of different WHO grades.** In order to obtain more objective data on KLF8 expression, cell counts of KLF8-immunopositive tumor cells were calculated in relation to the total number of cells within the tumor areas. There was no statistically significant difference in cell counts of KLF8-positive tumor cells of LGG (87%), AA (80%) and GBM (89%).
(TIF)

Figure S1 **Positive controls for KLF8 immunohisto-/immunocytochemistry.** (A) Due to their known high expression of KLF8 and in accordance with the manufacturerś guide, tissue samples from a renal parenchyma served as a positive control for immunohistochemical procedures and calibration of overall staining intensity. Scale bar as indicated. (B) Untreated U87-MG were subjected to immunocytochemical staining for KLF8, which was displayed mainly in the nucleus of the cells as visualized by BCIP/NBT. Scale bar as indicated.
(TIF)
