# Supplementary figures and images for: Krüppel-Like Factor 8 (KLF8) Is Expressed in Gliomas of Different WHO Grades and Is Essential for Tumor Cell Proliferation
Source: PLoS One. 2012 Jan 19;7(1):e30429. doi: 10.1371/journal.pone.0030429 (PMC3261906; doi:10.1371/journal.pone.0030429)

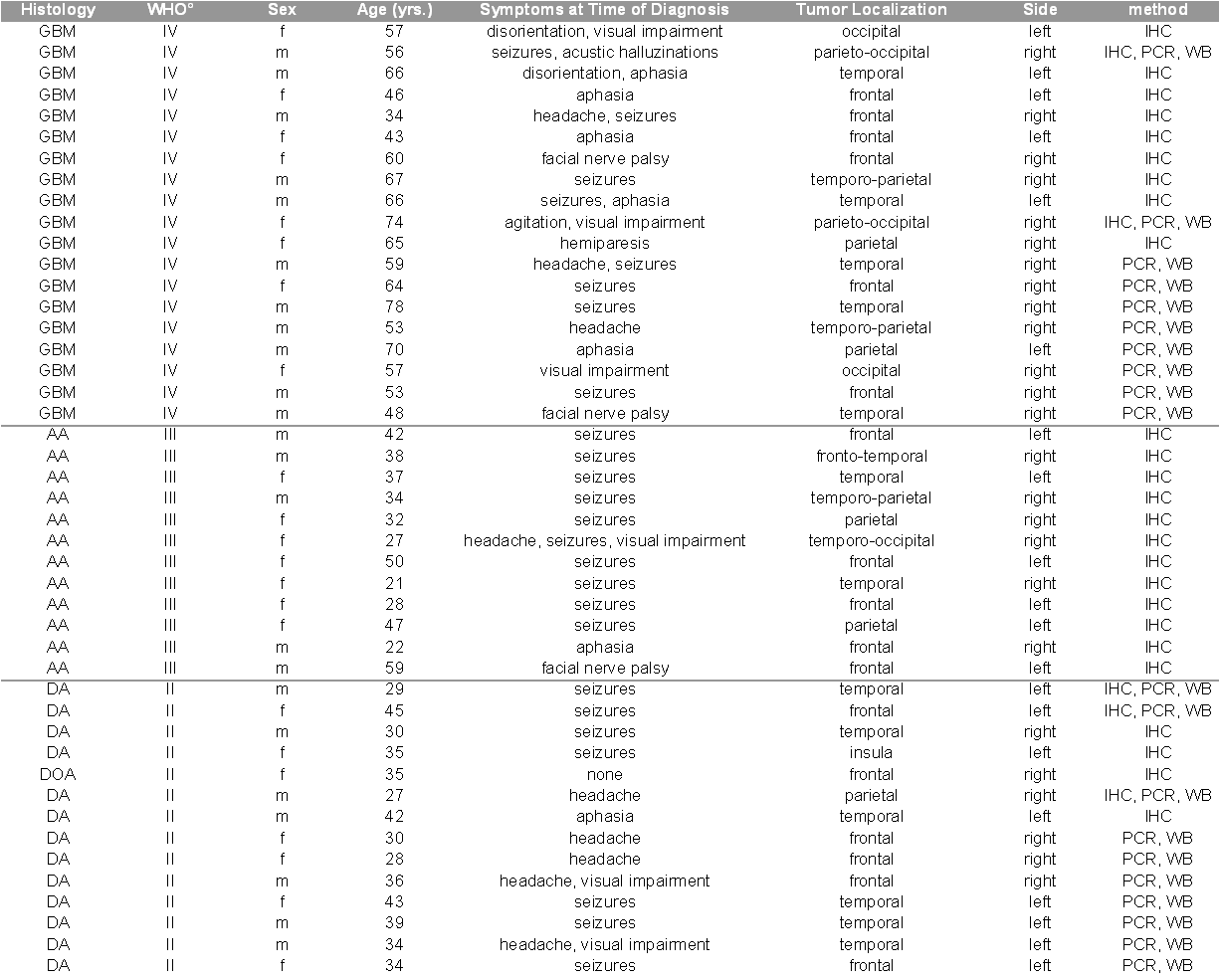

Supplement: Table S1 — Relevant clinical data from patients with gliomas of different WHO grades. Histologically confirmed low-grade astrocytomas (LGG; WHO°II), anaplastic astrocytomas (AA, WHO°III) and glioblastomas (GBM; WHO°IV) from both genders (male/female) were included in our study. Tumor tissue was subjected to the different analysis as indicated: IHC = immunohistochemistry, WB = Western blot, PCR = polymerase chain reaction. (TIF) [file pone.0030429.s001.tif]

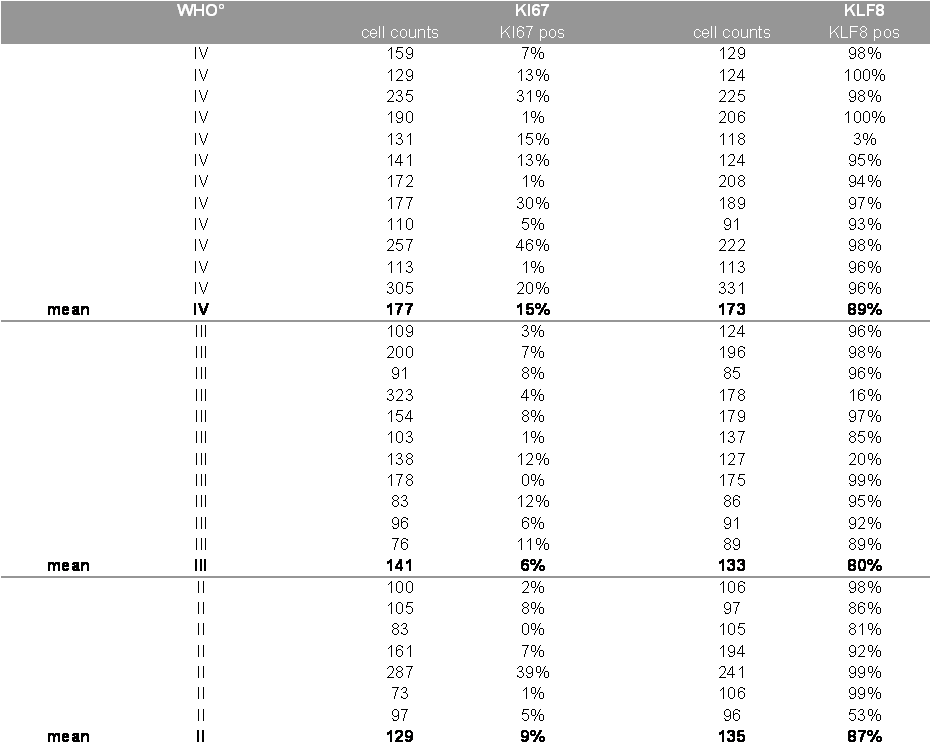

Supplement: Table S2 — Cell count analysis of KLF8 and Ki67 in gliomas of different WHO grades. In order to obtain more objective data on KLF8 expression, cell counts of KLF8-immunopositive tumor cells were calculated in relation to the total number of cells within the tumor areas. There was no statistically significant difference in cell counts of KLF8-positive tumor cells of LGG (87%), AA (80%) and GBM (89%). (TIF) [file pone.0030429.s002.tif]

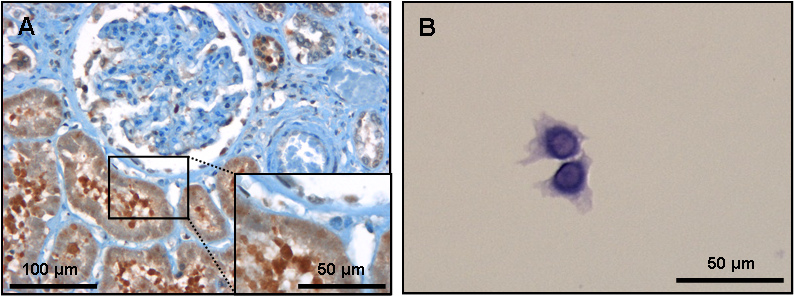

Supplement: Figure S1 — Positive controls for KLF8 immunohisto-/immunocytochemistry. (A) Due to their known high expression of KLF8 and in accordance with the manufacturerś guide, tissue samples from a renal parenchyma served as a positive control for immunohistochemical procedures and calibration of overall staining intensity. Scale bar as indicated. (B) Untreated U87-MG were subjected to immunocytochemical staining for KLF8, which was displayed mainly in the nucleus of the cells as visualized by BCIP/NBT. Scale bar as indicated. (TIF) [file pone.0030429.s003.tif]
